# Selection of *Sclerodermus pupariae* Reference Genes for Quantitative Real-Time PCR

**DOI:** 10.3390/insects16030268

**Published:** 2025-03-04

**Authors:** Ting Zhou, Huahua Feng, Jie Zhang, Yanlong Tang, Xiaoling Dong, Kui Kang

**Affiliations:** 1 MARA Key Laboratory of Sustainable Crop Production in the Middle Reaches of the Yangtze River (Co-Construction by Ministry and Province), College of Agriculture, Yangtze University, Jingzhou 434025, China; 18285815048@163.com (T.Z.); 18311642924@163.com (H.F.); 2 College of Biology and Agriculture, Zunyi Normal University, Zunyi 563006, China; 18886207491@163.com (J.Z.); 15120086160@163.com (Y.T.); 3 Biomedical Research Institute, Hubei University of Medicine, Shiyan 442000, China

**Keywords:** *S. pupariae*, reference gene screening, qRT-PCR, developmental stages

## Abstract

*S. pupariae* is a newly discovered species of parasitoid wasps. Research into its development, behavioral genetics, and molecular mechanisms provides valuable insights for improving integrated pest management strategies. Quantitative real-time PCR (qRT-PCR) is the most commonly used method for analyzing gene expression. This method requires the identification of stable reference genes to accurately evaluate transcriptional level variations. In this study, eight candidate reference genes (*TUB*, *TBP*, *RPS18*, *GAPDH*, *18S rRNA*, *RPL32*, *Actin*, and *EF1-α*) were identified and evaluated for their suitability as reference genes. Gene expression levels across different developmental stages were analyzed using three software tools, GeNorm, NormFinder, and BestKeeper, and the online tool RefFinder. The overall ranking of reference gene stability was as follows: *RPS18* > *18S rRNA* > *RPL32* > *GAPDH* > *Actin* > *TUB* > *TPB* > *EF1-α*. Ultimately, *RPS18* was determined to be the most stable reference gene.

## Simple Summary

The parasitoid wasp *Sclerodermus pupariae* exhibits strong active attack capabilities and is widely used in the control of wood-boring pests such as longhorn beetles and jewel beetles. Using three software tools and the online platform RefFinder, the expression levels of eight candidate reference genes were analyzed across different developmental stages to identify the most stable reference gene. The stability of the candidate reference genes was further validated under different temperature conditions. The *RPS18* gene was identified as the most suitable reference gene for *S. pupariae*, providing a reliable molecular foundation for future molecular studies of this species. This can be used for the subsequent validation of standardized target gene expression in various tissue preparations and samples at each developmental stage, while also providing an important resource for studying the relevant biological mechanisms of *S. pupariae*.

## 1. Introduction

In recent years, forestry pest problems have become increasingly prominent, especially wood-boring pests, which have caused significant economic losses worldwide [1]. Due to the concealed lifestyle of these pests, chemical pesticides often fail to achieve effective pest control. Utilizing natural enemy insects for pest control has been recognized domestically and internationally as an effective strategy. Parasitoid wasps exhibit high host specificity and have been widely used in biological pest control [2]. *Sclerodermus pupariae* Yang et Yao (Hymenoptera: Bethylidae) was initially discovered in Guangang Forest Park, Tianjin, China [3]. It is an ectoparasitoid of the prepupae and pupae of *Agrilus planipennis* [4]; the adult resembles an ant, with pronounced sexual dimorphism, and exhibits parthenogenesis [5]. *S. pupariae* shows strong host-seeking and active attacking capabilities. It parasitizes wood-boring pests, including longhorn beetles, jewel beetles, and bark beetles, during their feeding stages [6]. Unlike other species in the genus, female *S. pupariae* can fly in the wild, which enhances their dispersal rate and activity range. This trait makes *S. pupariae* a highly advantageous and promising tool for controlling wood-boring pests [7].

Parasitoid Hymenoptera, the most species-rich order of insects, can suppress host populations. For example, the specialist aphid parasitoid *Aphidius gifuensis* (Hymenoptera: Braconidae) is used to control the green peach aphid *Myzus persicae* [8], and the larval stages of the diamondback moth serve as natural hosts for the endoparasitoid wasp *Cotesia vestalis*. Many endoparasitoid wasps kill their hosts by producing effector molecules that inhibit growth and immune defenses [9]. Because its genome has not been reported, molecular studies on *S. pupariae* remain limited. Elucidating the molecular mechanisms of *S. pupariae* not only enhances its pest control efficiency but also provides theoretical insights into wing differentiation mechanisms in insects and practical implications for managing wood-boring pests.

In molecular biology, qRT-PCR is commonly used to measure gene expression. Reference genes are essential for detecting changes in target gene expression [10], helping to control internal differences and reduce errors between samples [11]. qRT-PCR is a highly sensitive, simple, and cost-effective molecular technique commonly used for quantifying gene expression levels [12]. Accurate gene expression analysis requires normalization using relatively stable reference genes, which show minimal expression differences across tissues and physiological states [13]. However, reference gene expression levels may vary under different experimental conditions [14]. Typically, qRT-PCR reference genes are involved in basic cellular functions, allowing for stable expression unaffected by external conditions [15]. The reference genes of *Drosophila*, *Bombus terrestris*, *Apis mellifera*, and *Tribolium castaneum* have been extensively validated and studied. Among them, three commonly used reference genes—elongation factor 1-alpha (Ef1-α), glyceraldehyde-3-phosphate dehydrogenase (*GAPDH*), and ribosomal protein L32 (*RPL32*)—have been shown to be stably expressed in the honeybee *Apis mellifera* [16]. Housekeeping genes such as ribosomal protein S18 (*18S rRNA*), elongation factor 1-alpha (Ef1-α), glyceraldehyde-3-phosphate dehydrogenase (*GAPDH*), RPSs, ribosomal protein L (*RPLs*), and beta-tubulin (*TUB*) are commonly used as reference genes [17]. In *S. pupariae*, different developmental stages and treatments can significantly influence gene expression. Therefore, selecting an appropriate reference gene is crucial.

Before analyzing gene expression, it is necessary to evaluate and identify the optimal reference gene for the experiment [18]. Candidate reference genes can be evaluated using algorithms provided by software tools such as GeNorm, NormFinder, and BestKeeper, which assess gene stability based on the variance in cycle threshold (Ct) values under various physiological or experimental conditions [19]. GeNorm screens stable reference genes by calculating the M value, where a smaller M value indicates higher stability [20]. NormFinder evaluates and selects reference genes by calculating the stability value of each gene and combining it with a model-based statistical approach; the smaller the stability value, the more stable the expression of the reference gene [21]. BestKeeper assesses stability by calculating stability parameters [22]. RefFinder is a web-based comprehensive algorithm that integrates the three main algorithms above. By comparison, it assigns different rankings to the analyzed reference genes, with candidate genes with lower mean weights being considered the most stable genes [18]. Using gene expression analysis tools can reveal the varying responses of specific genes to different environmental conditions, enabling us to infer the impact of genes on the survival, development, and reproduction of organisms.

The present study evaluates eight candidate reference genes (*TUB*, *TBP*, *RPS18*, *GAPDH*, *18S rRNA*, *RPL32*, *Actin*, *EF1-α*) in *S. pupariae* across different developmental stages and treatments. Multiple algorithms were used to identify the most stable and suitable reference gene, providing a solid foundation for future research on gene function, developmental regulation, and the biological and ecological adaptations of *S. pupariae.*

## 2. Materials and Methods

### 2.1. Insect Source

The population of *S. pupariae* was collected from Guangang Forest Park, Tianjin, China (38°56′ N, 117°29′ E). The wasps were reared in the laboratory using larvae of Thyestilla gebleri as hosts. The host larvae, weighing 230 ± 10 mg, were purchased from the Tianjin Flower, Bird, and Fish Market. Rearing conditions for the wasps were set at 24 ± 1 °C or 30 ± 1 °C, with a relative humidity of 55–65% and a photoperiod of L = 8:16 h.

### 2.2. Sample Collection and Treatment

Under 24 °C conditions, samples of *S. pupariae* at different developmental stages were collected, including 3rd to 5th instar larval (3 larvae per sample), pupae (9 individuals per sample), and newly emerged males and females (3 individuals per sample). Each stage was sampled in triplicate for biological replication. All samples were stored at −80 °C for subsequent RNA extraction.

### 2.3. RNA Extraction and cDNA Synthesis

Total RNA was extracted using a magnetic-column-based RNA rapid extraction kit (MIKX, MKG962-L, Shenzhen, China) following the manufacturer’s instructions. RNA concentrations were measured using a NanoDrop 2000 UV spectrophotometer (Thermo Fisher Scientific, Waltham, MA, USA). First-strand cDNA was synthesized from 1 μg of total RNA using the HiSlidTM cDNA Synthesis Kit for qPCR (with dsDNase) (MIKX, MKG840, Shenzhen, China) according to the manufacturer’s protocol. cDNA was stored at −20 °C for subsequent qRT-PCR analysis.

### 2.4. Primer Design and Quantitative Real-Time PCR

Eight pairs of candidate reference genes were selected based on commonly used reference genes in Hymenoptera insects: *EF1-α*, *RPS18*, *RPL32*, *GAPDH*, *18S rRNA*, *TUB*, *TBP*, and *Actin*. The gene sequences we obtained were predicted through the genome of *S. alternatusi* [23] and transcriptomics of *S. pupariae* [data not published] and then accurately sequenced following PCR amplification and using blastx in the National Center for Biotechnology Information (https://www.ncbi.nlm.nih.gov/, accessed on 5 September 2024). Primers were designed using Primer5 software and synthesized by Shanghai Bioengineering Co., Ltd., Shanghai, China, (Table S1). The qRT-PCR reactions were conducted using the 2 × Polarsignal qPCR Mix (MIKX, MKG800-10, Shenzhen, China) in a reaction volume of 20 μL, including 10 μL of 2 × Polarsignal qPCR Mix, 1 μL each of forward and reverse primers (10 μM), 1 μL of cDNA template, and 7 μL of sterile double-distilled water. Amplification was performed on a CFX Connect real-time PCR system (Bio-Rad, Hercules, CA, USA) under the following conditions: pre-denaturation at 94 °C for 20 s, followed by 40 cycles of 94 °C for 10 s and 60 °C for 20 s. After amplification, melting curve analysis was performed to verify primer specificity. Standard curves were constructed using tenfold serial dilutions of cDNA, and PCR efficiency and correlation coefficients (R^2^) were calculated from the slope of the curves.

### 2.5. Reference Gene Screening

The stability of the eight candidate reference genes under different treatments was evaluated using three software tools: GeNorm (version 2002), NormFinder (version 20), and BestKeeper (version 1). ΔCt values were calculated by subtracting the minimum Ct value (highest expression) of each gene across all samples from the Ct values of other samples. The resulting 2^−ΔCt^ values were used for GeNorm and NormFinder analyses [17]. GeNorm analysis: Stability (M) values were calculated for each gene, with lower M values indicating greater stability. Pairwise variation (V_n_/V_n+1_) was also calculated to determine the optimal number of reference genes required. A threshold of 0.15 was used; if V_n_/V_n+1_ < 0.15, the optimal number of reference genes is n; otherwise, it is n + 1 [19]. NormFinder analysis: stability values were calculated for each gene, with smaller values indicating more stable expression [20]. BestKeeper analysis: Stability was assessed using raw Ct values to calculate the correlation coefficient (r), standard deviation (SD), and coefficient of variation (CV). Genes with higher r, and lower SD and CV values, were considered more stable. Genes with SD > 1 were deemed unstable [21,22]. RefFinder integration: RefFinder, an online tool, combined the results from GeNorm, NormFinder, and BestKeeper to assign weighted rankings to the candidate genes, providing a comprehensive stability ranking [18].

### 2.6. Validation of Reference Gene Stability

To validate the stability of the selected reference gene, *S. pupariae* larvae reared at 24 °C and 30 °C were injected with 40 nL of dsInR solution (5 ng/nL, 200 ng/individual) [24] at the lateral abdominal segment using a microinjection device. Non-injected larvae served as controls. Samples were collected at 24, 48, and 72 h post-injection for qRT-PCR analysis.

### 2.7. Statistical Analyses

Relative gene expression levels were calculated using the 2^−ΔΔCt^ method, and statistical analyses were performed using SPSS Statistics 27.0. Significance was determined using Duncan’s test, and GraphPad Prism 10.3 was used for data visualization.

## 3. Results

### 3.1. Validation of Primer Specificity for Candidate Reference Genes

PCR products from the eight candidate reference genes showed single bands without primer dimers, and the band sizes matched the expected sizes (Figure S1A). qRT-PCR results revealed single melting peaks for all genes (Figure S1B). Amplification efficiencies ranged from 91% to 106%, meeting the required range of 90–110%. The correlation coefficients (R^2^) ranged from 0.9842 to 0.9985, exceeding the standard threshold of 0.9800 (Table 1). These results demonstrate that the selected primers exhibit high specificity and are suitable for qRT-PCR analysis.

### 3.2. GeNorm Analysis

**Figure 1:**
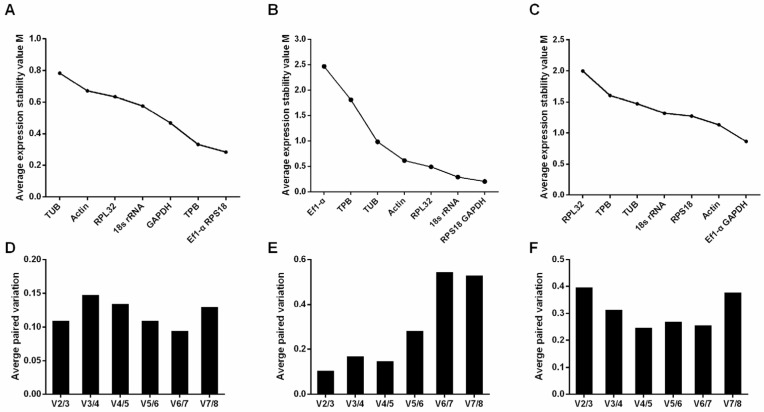
Stability ranking of candidate reference genes using GeNorm in *S. pupariae.* (**A**) M values of candidate reference genes in larval (3rd to 5th); (**B**) M values of candidate reference genes in prepupal stage, middle pupal stage, postpupal stage; (**C**) M values of candidate reference genes in male and female adult day one; (**D**) in larval (3rd to 5th) paired variation; (**E**) in prepupal stage, middle pupal stage, and postpupal stage paired variation; (**F**) in newly emerged males and females paired variation.

GeNorm calculates M values to rank reference gene stability, where lower M values indicate higher stability [18]. The results revealed significant differences in stability among the eight candidate genes across developmental stages and sexes (Figure 1). In instar larval: The two most stable genes were *RPS18* and *EF1-α*, while *TUB* and *Actin* were the least stable (Figure 1A). Pairwise variation analysis indicated that all variation values (V_n_/V_n+1_) were below 0.15, suggesting that *RPS18* and *EF1-α* are the most stable reference genes in the larval stage (Figure 1D). In prepupal stage, middle pupal stage, and postpupal stage: *RPS18* and *GAPDH* showed the highest stability (Figure 1B,E). In male and female adult: EF1-α and GAPDH were the most stable, and the M value of RPS18 was 1.27, slightly higher than EF1-α and GAPDH (0.86) (Figure 1C,F). Overall, *RPS18* was identified as the most stable gene based on GeNorm analysis.

### 3.3. NormFinder Analysis

NormFinder evaluates stability using a model-based statistical approach, ranking genes based on stability values (smaller values indicate higher stability) [19]. Larval stage: the stability rankings were *TBP* > *RPS18* > *EF1-α* > *Actin* > *18S rRNA* > *RPL32* > *GAPDH* > *TUB*, with TBP being the most stable and TUB the least (Table 2). Pupal stage: the rankings were *RPS18 = GAPDH* > *18S rRNA* > *Actin* > *RPL32* > *TUB* > *TBP* > *EF1-α*, with *RPS18* and *GAPDH* being the most stable. Adult stage: the rankings were *EF1-α* > *GAPDH* > *RPS18* > *18S rRNA* > *TUB* > *Actin* > *TBP* > *RPL32*, with *EF1-α* being the most stable (Table 2).

### 3.4. BestKeeper Analysis

BestKeeper ranks reference genes based on standard deviation (SD) and the coefficient of variation (CV). Genes with lower SD and CV values are considered more stable [20]. Larval stage: the stability rankings were *TUB* > *Actin* > *TBP* > *RPL32* > *EF1-α* > *RPS18* > *GAPDH* > *18S rRNA*, with *18S rRNA* showing the highest stability (Table 3). Pupal stage: *RPL32* was the most stable gene. Adult stage: *RPL32* was again the most stable (Table 3).

### 3.5. Comprehensive Analysis and Ranking of Candidate Reference Genes

The three software tools—GeNorm, NormFinder, and BestKeeper—use different algorithms to rank reference gene stability, which can lead to variations in their results. To provide a more comprehensive evaluation, the rankings from these tools were integrated using the online platform RefFinder. By assigning weighted scores to each gene based on the results of the three algorithms, RefFinder generated an overall ranking [16]. The final stability ranking of the reference genes was as follows: *RPS18* > *18S rRNA* > *RPL32* > *GAPDH* > *Actin* > *TUB* > *TBP* > *EF1-α*. These results suggest that *RPS18* is the most stable reference gene across all experimental conditions and developmental stages.

### 3.6. Validation of Reference Gene Stability

**Figure 2:**
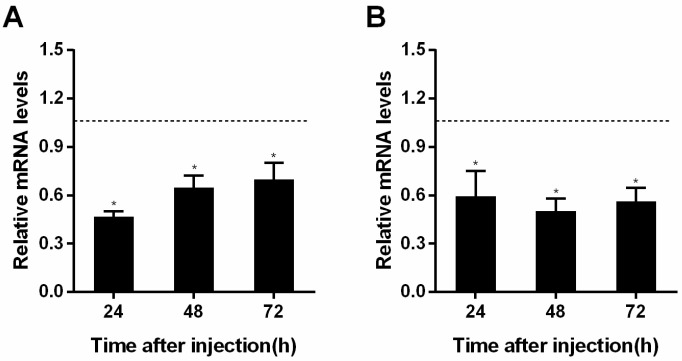
The target gene mRNA expressional level after injection of dsInR at different times. (**A**): 24 °C; (**B**): 30 °C. Each point represents the mean ± S.E. of three replicates. *, *p* < 0.05.

*RPS18*, identified as the most stable gene, was validated across different temperatures (24 °C and 30 °C) by injecting *S. pupariae* larvae with dsInR. Gene expression was measured at 24, 48, and 72 h post injection. The interference effect was significant at both temperatures. At 24 °C, the interference efficiency of InR reached 72.6% at 24 h (Figure 2A). At 30 °C, the interference efficiency peaked at 52.4% at 48 h (Figure 2B). These results confirmed that *RPS18* exhibits stable expression under different temperature conditions, making it a reliable reference gene for further molecular studies on *S. pupariae*.

## 4. Discussion

Gene expression analysis has become a cornerstone of biological research, and qRT-PCR is the preferred method due to its high sensitivity, accuracy, specificity, and rapidity [25]. However, experimental factors such as RNA integrity, reverse transcription efficiency, and cDNA quality [26] can introduce variability into gene expression data [17]. Reference genes are essential for normalizing such variability, ensuring the reliability of qRT-PCR results [27]. Reference genes must exhibit stable expression across different developmental stages, tissues, and experimental conditions [28]. Using stably expressed reference genes as internal controls is one of the most common methods for data normalization and is also an essential part of gene expression analysis [29]. Therefore, verifying the stability of reference gene expression is crucial for analyzing gene expression. In insect molecular biology, gene expression analysis is widely applied, primarily to study the cellular mechanisms in different developmental stages, tissues, and biological samples of insects [30]. Commonly used reference genes in insects include *18S rRNA*, *EF1-α*, *RPL18*, *RPS18*, *β-actin*, and *GAPDH*. In Hymenopteran insects, *RPL32*, *RPS5*, *RPS18*, and *TPB-αf* are frequently used as reference genes in studies related to their growth and development [13]. Research has shown that *RPS18* and *GAPDH* are stably expressed in *Apis mellifera* after infection with *Escherichia coli* [31]. Additionally, *RPS3*, *RPS18*, and *RPL13α* are stably expressed in *T. castaneum* after infection with *Beauveria bassiana* [32]. Studies also indicate that the *GAPDH* gene is stably expressed in the brain of worker bees (*Apis mellifera*) throughout their adult stage, facilitating research on the division of labor in *Apis mellifera* based on changes in gene expression [16].

To evaluate and screen reference genes for gene expression analysis, several computational programs have been developed over the past decade, including GeNorm, NormFinder, and BestKeeper [30]. The different algorithms used by these programs rank the stability of reference genes in varying ways, resulting in discrepancies in stability scores and rankings among them. RefFinder integrates the ranking results from all these algorithms, assigns appropriate weights to each gene, and calculates the geometric mean of the gene weights to obtain a final comprehensive ranking. Candidate genes with lower average weights are considered stable and can be used as ideal reference genes [33]. In this study, GeNorm, NormFinder, and BestKeeper were used to evaluate the stability of eight candidate reference genes in *S. pupariae*. Although the most stable genes slightly vary in terms of conditions and analysis methods, our results indicate that in qRT-PCR analysis, RPS18 consistently demonstrated the highest stability across developmental stages and experimental conditions. RefFinder further confirmed *RPS18* as the most reliable reference gene. RNA injection may have an impact on its physiology. We used this method to verify whether the expression of *RPS18* remains stable after injecting the dsInR gene. At both 24 °C and 30 °C, *RPS18* showed stable expression, with consistent Ct values ranging from 15.31 to 16.87 (Table S2). Environmental temperature is a key factor influencing insect growth, development, and reproduction. For example, the reproductive rate of the white-backed planthopper (*Sogatella furcifera*) is higher at lower temperatures, while the brown planthopper (*Nilaparvata lugens*) exhibits higher reproductive rates at higher temperatures [34]. Similarly, temperature significantly affects the development, colony activity, individual behavior, and disease resistance of honeybees (*Apis mellifera*) [35]. Reference genes are used as internal controls due to their relatively stable expression levels, which allow for data normalization. qPCR data normalization is performed using the quantification cycle (Cq) value, which is defined as the cycle at which the fluorescence level reaches a threshold that can be manually or automatically set [36]. Despite variations in the tissues, developmental stages, or physiological conditions of the evaluated species, the expression levels of these genes remain constant [37]. Stable reference genes like *RPS18* allow for accurate normalization of qRT-PCR data under varying temperature conditions, making them indispensable for gene expression studies.

Regarding insects, numerous studies have been conducted to evaluate the selection and validation of reference genes under various biotic and abiotic conditions. In these studies, different types of housekeeping genes were chosen for the analysis of gene expression stability [38]. Housekeeping genes are essential for maintaining cellular structure or function, and their expression levels are relatively stable compared to tissue-specific genes [39]. Among them, actin, ribosomal protein 49 (*RP49*), and elongation factor 1-α are stably expressed in *Apidae mellifera* [40]. Similarly, ribosomal protein genes *RPL32*, *RPS5*, *RPS18*, and *TPB-αf* have shown the highest stability in three stingless bee species [12]. Research has demonstrated that *RPL13* and *RP49* genes are stably expressed during different developmental stages of bumblebees (*Bombus terrestris*), which can enhance the accuracy of gene expression analysis across developmental stages in this species [41]. Meanwhile, in real-time quantitative analyses of different bee tissue samples collected across various seasons, the genes *RPS18* and *GAPDH* were identified as the most suitable reference genes for bees [42]. Additionally, *RPS18* was found to be the most stable reference gene for analyzing gene expression patterns in *Polyphagotarsonemus* latus populations exposed to acaricides and temperature stress [43]. Previous studies have shown that *RPS18* is stably expressed across four different strains and developmental stages of *Tetranychus cinnabarinus* (Subclass Acari: Family Tetranychidae) [44]. Experimental results indicate that in *S. guani* reared under different temperature conditions, the *RPS18* gene is unaffected by temperature and exhibits extremely high stability. Research has revealed that ribosomal protein genes exhibit high stability under various biotic and abiotic conditions in insects [40]. The ribosomal protein S genes (*RPS3*, *RPS11*, *RPS15*, and *RPS18*) demonstrate high stability during different developmental stages of the housefly (*Musca domestica*) [38], the brown planthopper (*Nilaparvata lugens*) [45], and Coleopteran insects [46]. These genes also exhibit high stability across different tissues and under abiotic stress conditions. Similarly, ribosomal protein L genes (*RPL18* and *RPL32*) have been identified as highly stable in the tissues of the red imported fire ant (*Solenopsis invicta*) [14] and *N. lugens* [45], with consistently high expression stability under abiotic stress conditions. Most ribosomal protein (R-protein) genes are organized within highly conserved operons, and in many cases, their expression is regulated through feedback mechanisms, where one or more protein products of a given operon act as regulators. Once the binding sites on ribosomal RNA (rRNA) are saturated, the regulatory proteins bind to their own mRNA, often in the 5′ untranslated region (5′ UTR) [47]. Ribosomal proteins (RPS) are essential components of the ribosome, the universally conserved machinery that translates genetic information into proteins. The labeled *RPS18* and *RPL11* can complement genetic deficiencies in the corresponding genes [48]. *RPS18* is a newly identified PGN-binding protein, abundantly present in the eggs and embryos of zebrafish. Recombinant RPS18 can bind to bacteria as a pattern recognition receptor and it kills both Gram-positive and Gram-negative bacteria as an effector molecule of the immune response [49]. The combination of *RPS18* and *RPL13* can be used as reference genes for each experimental condition at different developmental stages, tissues, and temperatures of *Henosepilachna vigintioctopunctata* [50]. In *Aphidius gifuensis*, *RPS18* is the most stable reference gene in different sexes and tissues [51]. In the context of bacterial infection, the head *RPS18* and *GAPDH* genes of *Apis mellifera* L. (Hymenoptera: Apidae) are able to express stably [52].

## 5. Conclusions

*RPS18* has been identified as the most stable reference gene in *S. pupariae* across different developmental stages and temperature conditions. This study provides a reliable foundation for subsequent molecular studies on this parasitoid wasp and offers significant insights for its practical application in wood-boring pest control. The findings highlight the importance of selecting appropriate reference genes for accurate normalization in gene expression studies, ensuring robust and reliable results.

## Figures and Tables

**Table 1:** qRT-PCR standard curves of five candidate reference genes in *Sclerodermus pupariae*.

Gene	R^2^	Efficiency (%)
*EF1-α*	0.9842	93
*RPS18*	0.9859	101
*RPL32*	0.992	91
*GAPDH*	0.9985	92
*18s rRNA*	0.9922	106
*TUB*	0.9985	93
*TPB*	0.9989	100
*Actin*	0.9829	91

**Table 2:** Stability ranking of candidate reference genes using NormFinde in *S. pupariae*.

Gene	Larva		Pupa		Adult	
	Stability Value	Stability Rank	Stability Value	Stability Rank	Stability Value	Stability Rank
*EF1-α*	0.236	3	2.904	7	0.299	1
*RPS18*	0.214	2	0.070	1	0.767	3
*RPL32*	0.387	6	0.611	4	2.067	8
*GAPDH*	0.492	7	0.070	1	0.698	2
*18s rRNA*	0.379	5	0.113	2	0.773	4
*TUB*	0.706	8	1.189	5	0.850	5
*TPB*	0.194	1	2.669	6	1.311	7
*Actin*	0.377	4	0.263	3	0.863	6

**Table 3:** Stability ranking of candidate reference genes using BestKeeper in *Sclerodermus pupariae*.

Gene	Larva			Pupa			Adult		
	SD	CV	Stability Rank	SD	CV	Stability Rank	SD	CV	Stability Rank
*EF1-α*	0.77	5.12	4	2.41	13.45	8	1.43	4.95	3
*RPS18*	0.72	4.51	3	0.49	2.84	4	1.87	7.59	5
*RPL32*	0.85	3.10	5	0.35	1.30	1	0.96	2.91	1
*GAPDH*	0.56	3.27	2	0.37	2.09	2	2.11	7.39	7
*18s rRNA*	0.43	3.60	1	0.42	3.47	3	1.41	7.06	2
*TUB*	0.99	5.08	8	1.76	7.99	6	1.63	5.08	4
*TPB*	0.88	3.79	6	2.36	9.73	7	1.99	6.33	6
*Actin*	0.96	3.13	7	0.82	2.71	5	2.12	7.08	8

## Data Availability

Data are contained within this article or the Supplementary Materials.

## Supplementary Materials

The following supporting information can be downloaded at: https://www.mdpi.com/article/10.3390/insects16030268/s1, Figure S1: Melting curve of qRT-PCR of candidate reference gene; Table S1: Primer sequence of candidate reference genes; Table S2: The cycle threshold (CT) of RPS18 in injection of dsInR at different temperatures.

## Abbreviations

The following abbreviations are used in this manuscript:

RPS18	Ribosomal Protein S18
qRT-PCR	Quantitative real-time PCR
TUB	Beta-tubulin
TPB	TATA-box binding protein
GAPDH	Glyceraldehyde-3-phosphate dehydrogenase
18S rRNA	Ribosomal protein S18
RPL32	Ribosomal protein L32
EF1-α	Elongation factor 1-α
Ct	Cycle threshold
InR	Insulin receptor
RPS5	Ribosomal protein S5
TPB-αf	TATA-box binding protein-αf
RPS3	Ribosomal Protein S3
RPL13α	Ribosomal protein L13α
Cq	Quantification cycle
RP49	Ribosomal protein 49
RPL13	Ribosomal protein L32
RPS11	Ribosomal Protein S11
RPS15	Ribosomal Protein S15
