# Supplementary material for: Selection of Sclerodermus pupariae Reference Genes for Quantitative Real-Time PCR
Source: Insects. 2025 Mar 4;16(3):268. doi: 10.3390/insects16030268 (PMC11943240; doi:10.3390/insects16030268)
Supplement: Supplementary file 1 [file insects-16-00268-s001.zip › Insects-3445947 Supplementary/Supplementary Figure S1.pptx]

## Slide 1
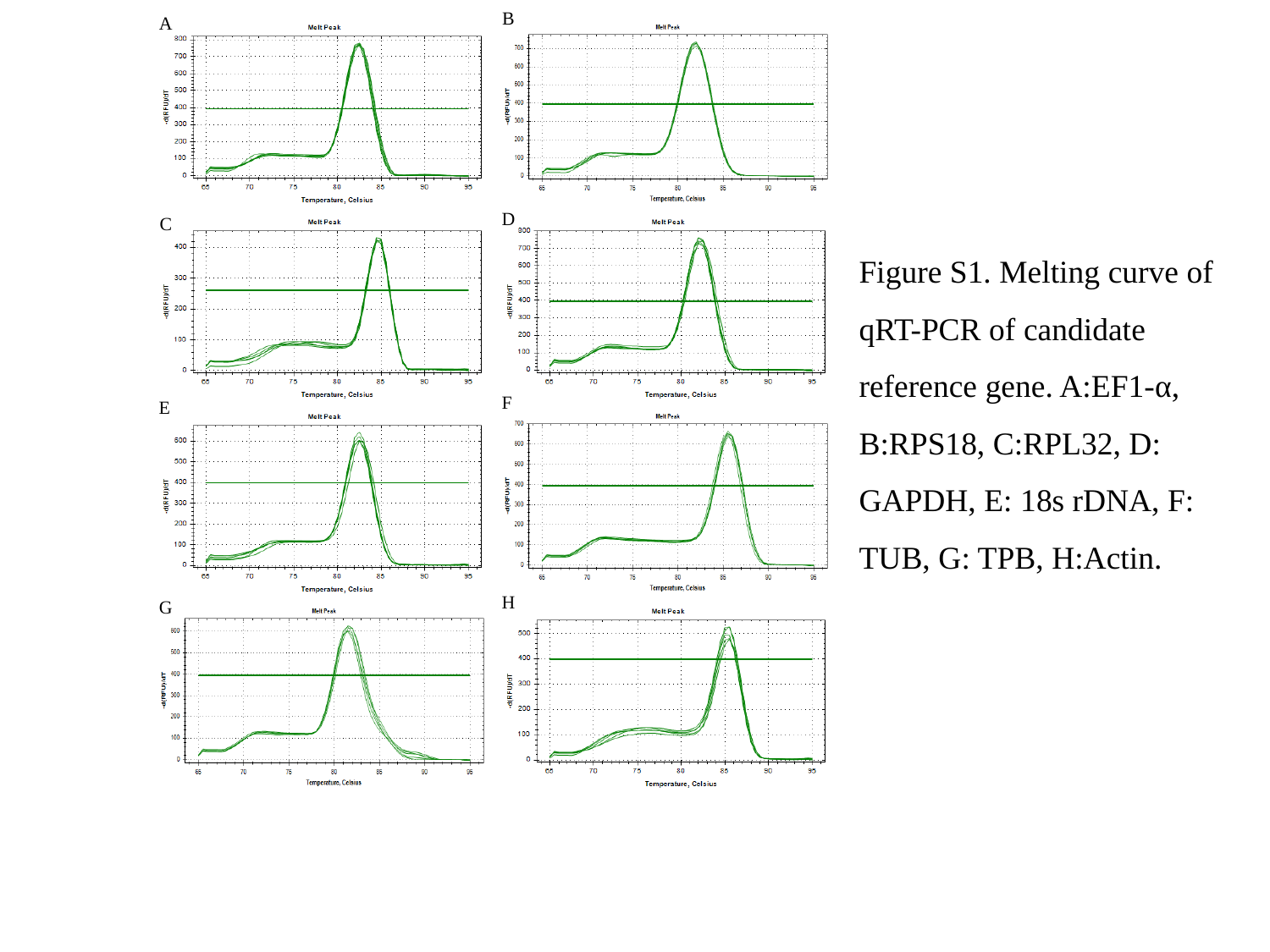

B
A
D
C
Figure S1. Melting curve of qRT-PCR of candidate reference gene. A:EF1-α, B:RPS18, C:RPL32, D: GAPDH, E: 18s rDNA, F: TUB, G: TPB, H:Actin.
F
E
H
G
